# Treatment with the NR4A1 agonist cytosporone B controls influenza virus infection and improves pulmonary function in infected mice

**DOI:** 10.1371/journal.pone.0186639

**Published:** 2017-10-20

**Authors:** Benoit Egarnes, Marie-Renée Blanchet, Jean Gosselin

**Affiliations:** 1 Laboratory of Innate Immunology, Centre de recherche du CHU de Québec-Université Laval (CHUL) and Department of Molecular Medicine, Université Laval, Quebec, QC, Canada; 2 Institut Universitaire de Cardiologie et de Pneumologie de Québec, Université Laval, Québec, QC, Canada; Louisiana State University, UNITED STATES

## Abstract

The transcription factor NR4A1 has emerged as a pivotal regulator of the inflammatory response and immune homeostasis. Although contribution of NR4A1 in the innate immune response has been demonstrated, its role in host defense against viral infection remains to be investigated. In the present study, we show that administration of cytosporone B (Csn-B), a specific agonist of NR4A1, to mice infected with influenza virus (IAV) reduces lung viral loads and improves pulmonary function. Our results demonstrate that administration of Csn-B to naive mice leads to a modest production of type 1 IFN. However, in IAV-infected mice, such production of IFNs is markedly increased following treatment with Csn-B. Our study also reveals that alveolar macrophages (AMs) appear to have a significant role in Csn-B effects, since selective depletion of AMs with clodronate liposome correlates with a marked reduction of IFN production, viral clearance and morbidity in IAV-infected mice. Furthermore, when reemergence of AMs is observed following clodronate liposome administration, an increased production of IFNs was detected in bronchoalveolar fluids of IAV-infected mice treated with Csn-B, supporting the contribution of AMs in Csn-B effects. While treatment of mice with Csn-B induces phosphorylation of transcriptional factors IRF3 and IRF7, the latter appears to be less indispensable since effects of Csn-B treatment on the synthesis of IFNs were slightly affected in IAV-infected mice lacking functional IRF7. Together, our results highlight the capacity of Csn-B and consequently of NR4A1 transcription factor in controlling IAV infection.

## Introduction

The transcription factor NR4A1, also named Nur77, has emerged as an important player of the immune response through its contribution to maintain homeostasis and its capacity to attenuate general inflammation [1, 2]. For example, it was demonstrated that NR4A1 may control inflammation by preventing translocation of NF-κB via the activation of the inhibitory protein IκB [3]. On the other hand, an increased phosphorylation of NF-κB was detected in NR4A1-deficient macrophages, supporting the contribution of NR4A1 in the control of inflammation [1]. Furthermore, mice deficient for NR4A1 present an enhancement of arteriosclerosis along with an increase of atherosclerotic lesions due to the differentiation of inflammatory Ly6C^high^ monocytes into macrophages displaying an M1 inflammatory phenotype. NR4A1 was also found to contribute to limit the influx of inflammatory monocytes and the production of inflammatory cytokines during myocardial infarction and NR4A1-dependent Ly6C^low^ monocytes have demonstrated a crucial role in mediating intravascular homeostasis by regulating necrosis of endothelial cells [4, 5]. These results are in line with the role of NR4A1 in the development of Ly6C^low^ patrolling monocytes which are recognized to be involved in the resolution of inflammation [6]. In addition, overexpression of NR4A1 in transgenic mice attenuates the severity of inflammation in a collagen-induced arthritis model [7].

Since its discovery, NR4A1 was initially believed to be regulated at post-translational levels such as phosphorylation and that no ligand recognition is required to activate NR4A1 [2, 8]. However, although no endogenous ligands of NR4A1 have yet been identified, pharmacological regulation of NR4A1 activity has been demonstrated with cytosporone B (Csn-B) [9]. Indeed, Csn-B physically binds to NR4A1 resulting in an increased expression and stimulation of its transcriptional activity *in vivo*. While little is known on the *in vivo* effects of Csn-B administration, recent studies reported the beneficial role of Csn-B in two distinct models of inflammatory diseases. Treatment with Csn-B was found to reduce the formation of atherosclerotic plaques in mice fed with a high-cholesterol diet, and to significantly alleviate the inflammation in colitis mouse model, in part due to a reduction of TNF-α and IL-6 synthesis [10, 11], suggesting that targeting NR4A1 may represent a potential target to control the inflammatory response.

Infection of host by pathogens like influenza virus (IAV) can lead to an excessive production of inflammatory mediators leading to an enhanced pulmonary inflammation and lung illness [12]. While IAV infection is not fatal for most people, elderly, children and those with chronic pulmonary diseases, such as asthma, are at high risk of developing severe complications [13, 14]. Despite the fact that seasonal vaccination campaigns have reduced the incidence and severity of influenza infection, a considerable threat for a pandemic as well as an enormous economic burden is still present [15, 16]. Only few drugs currently available have shown potential to control IAV infection. However, due to the high levels of mutation occurring in the most influenza strains, the development of new therapeutic strategies to better counter IAV infection is thus urgently needed. This prompted us to evaluate whether treatment of IAV-infected mice with the NR4A1 agonist Csn-B may control viral infection and reduce excessive lung inflammation. The results obtained show that treatment with Csn-B significantly reduces lung viral load and also improves pulmonary function. Depletion of alveolar macrophages (AMs) results in an impaired secretion of IFNs, supporting the essential role of AMs in Csn-B action. Together, our results provide evidence that Csn-B and consequently NR4A1 has the capacity to control IAV infection and to reduce excessive lung inflammation in infected mice.

## Materials and methods

### Ethics statement

This study was carried out in accordance with the recommendations of the Guide for the Care and Use of Laboratory Animals of the Canadian Council on Animal Care (CCAC). All protocols were approved by the Committee on the Ethics of Animal Experiments of Université Laval (Approval Number: 15-109-2).

### Mice

Four to six week old females C57Bl/6 wild-type (WT) mice were obtained from Charles River (St-Constant, Quebec, Canada) laboratories. Mice deficient for NR4A1 (*Nr4a1*^*-/-*^) were kindly provided by Dr. Claude Rouillard (Laval University, Quebec, Canada). *Irf3*^*-/-*^ mice were provided by Dr. Karen Mossman (McMaster University, Hamilton, Ontario, Canada) and *Irf7*^*-/-*^ mice were provided by Dr. Ian Rifkin (Boston University Medical Center, MA, USA). All mice colonies and littermates were housed in a specific pathogen-free facility at the Centre de Recherche du CHU de Quebec, Laval University.

### Viral infections

Infections were performed using Influenza virus (IAV) strain A/Puerto Rico/8/34 (H1N1). IAV was propagated and isolated from Madin-Darby canine kidney (MDCK) cells and titrated using standard plaque assay in MDCK cells as reported [17]. The MDCK cell line was cultured in MEM supplemented with 10% heat-inactivated FBS. Animals were infected intranasally (in.), with a sublethal dose of IAV (50 Plaque forming unit (PFU)) or otherwise indicated. We daily assessed the general health of the animals by monitoring their physical appearance, body weight and temperature. In the case where mice had lost more than 20% of their initial weight, they were sacrificed by lethal dose of isoflurane inhalation as recommended by the CCAC. In survival experiments following IAV infection, animals are not subjected to pain or distress and we did not observe any unexpected death. To determine lung viral loads, mice were sacrificed by lethal dose of isoflurane and lungs harvested at day 3, 5, 7 and 9 post-infection and viral burden was measured in lung homogenates using standard plaque assay in MDCK cells as described [17].

### *In vivo* treatment

Cytosporone B was purchased from Sigma-Aldrich (Oakville, ON, Canada) and reconstituted with DMSO. Drug was freshly dissolved in saline (0.9% p/v) prior to daily treatment of mice (5 mg/kg intraperitoneal, ip.). Treatment with Cytosporone B starts one day post IAV infection. Control mice were injected with placebo (0.9% saline, 3.5% DMSO v/v).

### *In vivo* depletion of alveolar macrophages

Mouse alveolar macrophages were depleted using intranasal administration of dichloromethylene-biphosphonate (clodronate)-loaded liposomes (100 μl) (Clodronate liposomes, Amsterdam, Netherlands), 24 hours prior to influenza virus infection [18]. Control animals received PBS-loaded liposomes. Alveolar macrophages depletion efficiency was monitored at indicated times by flow cytometry analysis.

### Flow cytometry analysis

Single-cell suspensions obtained from lungs homogenates were first incubated with anti-CD16/32 (clone 93 BioLegend, San Diego, CA, USA) to block non-specific antibody interaction with Fc receptors. Lungs alveolar macrophages were identified using anti-CD45 (clone 30F11; BD Biosciences, San Diego, CA, USA), anti-CD11b (clone M1/70; BD Biosciences), anti-F4/80 (clone BM8; BioLegend, San Diego, CA, USA), anti-Siglec F (E50-2440; BD Biosciences, San Diego, CA, USA) and anti-CD11c (clone HL-3; BD Biosciences, San Diego, CA, USA). A viability dye (LIVE/DEAD Fixable; Molecular Probes) was added to discriminate live cells. Autofluorescent cells were excluded by gating on CD45^+^and FITC^-^. Thereafter, total dendritic cells were identified with anti-CD11c, anti-CD11b, anti-IA/IE (clone M5/114.15.2, BD Biosciences), anti-CD45 and anti-F4/80. Flow cytometry was performed using BD LSR II (BD Biosciences, Ontario, Canada) and data analysed with FACSDiva software Version 6.1.2 (BD Biosciences, Ontario, Canada).

### Western blot analysis

Western blot analyses were performed on protein samples extracted from lung homogenates of WT and *Nr4a1*^*-/-*^ mice, infected or not with IAV (50 PFU) and treated with cytosporone B (5 mg/kg). Lungs were harvested at day 5 post-infection and samples were homogenized in ice-cold cell lysis buffer (Cell Signaling Technology, Danvers, MA, USA) containing protease and phosphatase inhibitor cocktail (Roche Applied Science, Laval, QC, Canada). Protein concentrations were determined by the BCA assay (Pierce, Rockford, IL, USA). Equal amounts of protein (40 μg) were separated on SDS/10% PAGE, transferred onto PVDF membranes and immunoblotted overnight with selected anti-phospho-IRF3 (Ser396) anti-phospho-IRF7 (Ser471/472) (Bioss, Worburn, MA, USA), anti-IRF3, anti-IRF7 (Santa Cruz, Dallas, TX, USA antibodies and anti-β actin (Biolegend, San Diego, CA, USA). Membranes were washed in Tris-buffered saline (TBS)-0.1% Tween 20 solution (Thermo Fisher Scientific, Rockford, IL, USA) prior to incubation with appropriate secondary horseradish peroxidase (HRP)-conjugated antibody (Jackson Immunoresearch, West Grove, PA, USA). HRP activity was revealed by incubation with the Clarity ECL substrate (Bio Rad, Mississauga, ON, Canada). Chemiluminescence reactions were visualized and quantitatively analyzed using Alphaview software (Alpha Innotech Corp., San Leandro, CA, USA).

### Histological analysis

WT and *Nr4a1*^*-/-*^ mice were infected or not with IAV (50 PFU) and daily treated with placebo (0.9% saline, 3.5% DMSO v/v) or cytosporone B (5 mg/kg). When indicated, WT mice were injected with clodronate liposomes 24 hours prior to IAV infection. Animal’s lungs were harvested and fixed in paraformaldehyde (4%). Tissues were embedded in paraffin and lungs sections were stained with Hematoxylin and Eosin (H&E) for histological analysis [19].

### RNA extraction, RT-PCR and qRT-PCR analysis

Total RNA was extracted using the QIAmp Viral RNA Mini Kit (Qiagen, Valencia, CA, USA), following the manufacturer’s instructions and DNatse-treated RNA (1 μg) was reverse-transcribed into cDNA using 40 units of RT Superscript II (Invitrogen) as previously detailed [20]. cDNA was used for amplification of IAV genes involved in viral replication using the following primers: matrix protein 1 (M1) [21]: (forward: 5’ -ATA TAC AAC AGG ATG GGG GCT- 3’ and reverse: 5’ -ATT TGC CTA TGA GAC CGA TGC T- 3’), IAV nonstructural protein (NS1) [22]: (forward: 5’ -GAT AGT GGA GCG GAT TCT GA- 3’ and reverse: 5’ -ATA CAA AGA GGG CCT GCC ACT-3’) and IAV polymerase basic 2 (PB2) [23]: (forward: 5’ -CGA GAT GTC AAT GAG AGG AGT G-3’ and reverse: 5’ -CCT CGT TGG TCC CGG ATT CT-3’).

Cells from bronchoalveolar lavages (BALs) were homogenized in TRIzol Reagent (Life Technologies, Burlington, ON, Canada) and RNA extraction was performed following manufacturer’s instructions. Extracted RNA was amplified using SYBR® GreenER^™^ qPCR SuperMix Universal (Life Technologies) with the following primers: to detect NR4A1 gene: forward 5’-CTG CGA AAG TTG GGG GAG T-3’ and reverse: 5’-CTT GAA TAC AGG GCA TCT CCA G-3’. cDNA was normalized with GAPDH using the following primers: forward: 5’-TGT TCC AGT ATG ACT CCA CTC ACG- 3’ and reverse: 5’ -ATG GTG GTG AAG ACA CCA GTA GAC- 3’.

### Measurement of IFN-β and IFN-α

IFN-β and IFN-α levels were determined in bronchoalveolar lavages (BALs) of WT, *Nr4a1*^*-/-*^, *Irf3*^*-/-*^ and *Irf7*^*-/-*^ mice infected or not with IAV (50 PFU) and treated with cytosporone B (5 mg/kg) or placebo. BALs fluids were collected at day 3, 5 and 7 following IAV-infection or otherwise indicated. IFN-β and IFN-α levels were determined using ProcartaPlex Mouse IFN alpha/IFN beta Panel (eBioscience, San Diego, USA). Samples were analyzed with a BD FACS CANTO II flow cytometer and interferons concentrations were determined with FCAP Array software (BD Biosciences). The limit of detection was 4 pg/ml.

### Measurement of cytokines

Levels of TNF-α and IL-6 were determined in lung homogenates of not infected or IAV infected WT mice treated with placebo or cytosporone-B by using the BD Cytometric Beads Array system (CBA Flex Set; BD Bioscience). Samples were analyzed with a BD FACS CANTO II flow cytometer and cytokines concentrations were determined with FCAP Array software (BD Biosciences).

### Analysis of pulmonary function

Mice were anesthetized with ketamine-xylazine (100 and 10 mg/kg, respectively), tracheotomized with an 18G catheter and connected to a computer-controlled ventilator (FlexiVent; SCIREQ, Montreal, QC, Canada [24, 25]). The respiratory frequency was set at 150 breaths/min, with a tidal volume of 10 ml/kg and a positive end-expiratory pressure of 3 cmH_2_O. Once ventilation was established, mice were paralyzed via an intramuscular injection of 0.1 mg/kg of pancuronium bromide. Increasing doses of methacholine (MCh) were delivered by nebulization during tidal breathing. Data obtained from single-frequency forced oscillation maneuvers were used to calculate overall respiratory system resistance and elastance, while a constant phase model was used to determine tissue damping and tissue elastance.

### Statistical analysis

All analyses were performed using Graph Pad Prism version 6.02 software (Graph Pad Software, San Diego, CA, USA). Statistical significance was set at p < 0.05. Differences in groups were determined with a two way analysis of variance (ANOVA) followed by a Tukey post-hoc test or otherwise indicated.

## Results

### Treatment with cytosporone B reduces lung viral load and improves survival in mice infected with Influenza A virus

**Fig 1:**
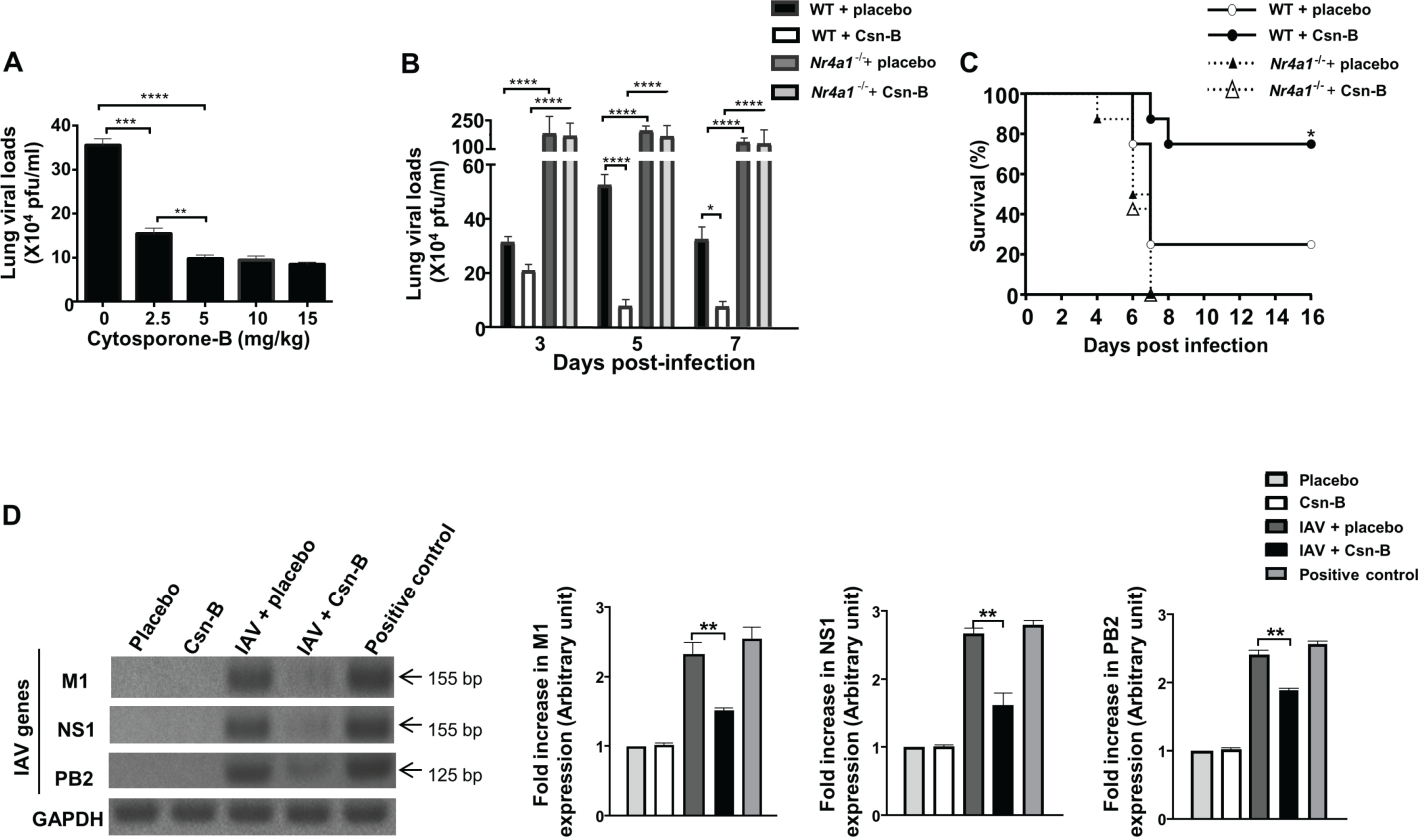
Treatment with cytosporone B reduces lung viral load and improves survival in mice infected with Influenza A virus. **(A)** Mice (n = 5/group) were infected with IAV and daily treated with placebo or with increasing concentrations of Csn-B administered intraperitoneally (ip.). Lungs were harvested at day 5 post IAV-infection (p.i.) for viral load determination. For lung viral loads, differences were analysed using One-Way ANOVA followed by Tukey post-hoc test. ** *p* ≤ 0.01, *** ≤ 0.001 and ****p ≤ 0.0001 as compared to indicated groups. **(B)** Lung viral loads were assessed in IAV-infected WT and *Nr4a1*^*-/-*^ mice (50 PFU), daily treated with placebo or Csn-B (5 mg/kg ip.). Lungs were harvested at day 3, 5 and 7 post-infection. Results are presented as mean ± SEM of two independent experiments (total of 10 mice/group). For lung viral loads, differences were analysed using Two-Way ANOVA followed by Dunnett post-hoc test. *p ≤ 0.05 and ****p ≤ 0.0001 as compared to indicated groups. **(C)** WT and *Nr4a1*^*-/-*^ mice (n = 8/group) were infected with IAV (3000 PFU in.) and daily treated for 16 days with either placebo or Csn-B (5 mg/kg). Survival was monitored daily. Differences were analysed using a log rank test (*p≤0.05 as compared to WT mice treated with a placebo). **(D)** Amplification of IAV genes (M1, NS1 and PB2) was performed by RT-PCR at day 5 on lung homogenates of IAV-infected mice treated with Csn-B or placebo. bp: base pair. Positive control: MDCK cells infected with IAV (4000 PFU/ml). Fold increase of gene expression is expressed relative to the placebo group. Data are representative of two independent experiments (n = 5mice/group). Differences were analysed using One-Way ANOVA followed by Tukey post-hoc test. ** *p* ≤ 0.01 as compared to indicated groups.

Firstly, we have assessed by RT-PCR specificity of Csn-B for NR4A1 in lungs of mice following five days of treatment. We found that Csn-B administration increases expression of NR4A1 mRNA but has no effect of NR4A2 and NR4A3 mRNA levels, supporting the specificity of Csn-B as an agonist of NR4A1 [9] (S1A Fig). Next, in order to determine whether Csn-B treatment can control IAV infection, infected mice were treated daily with increasing doses of NR4A1 agonist post-infection and lung viral loads were assessed at day 5 post-infection, which corresponds to the highest replicative activity of IAV [26]. As shown in Fig 1A, optimal effects of Csn-B treatment in reducing lung viral loads were obtained with the administration of 5 mg/kg. No significant reduction in viral loads was measured when using higher doses of Csn-B. Kinetics of the effects of Csn-B showed a drastic decrease at day 5 and 7 pi., as compared to the placebo-treated controls (Fig 1B). As expected, in mice deficient for NR4A1 (*Nr4a1*^*-/-*^), effects of Csn-B were totally abolished and a marked increase of lung viral loads was detected. We also showed a significant improvement of survival in IAV-infected mice treated with Csn-B compared to control animals (Fig 1C). Again, Csn-B treatment has no effect on IAV-infected *Nr4a1*^*-/-*^ mice. In order to determine whether such effects of Csn-B also correlate with a reduction of specific genes involved in IAV, we have looked at levels of mRNA expression of M1, NS1 and PB2 IAV genes by RT-PCR [27]. Interestingly, mRNA expression of all three genes was significantly reduced in mice following treatment with Csn-B (Fig 1D). Collectively, these results show that triggering of NR4A1 with Csn-B significantly contributes to protect mice from acute influenza infection.

### Treatment with cytosporone B improves lung structure, and functions in mice infected with Influenza A virus

**Fig 2:**
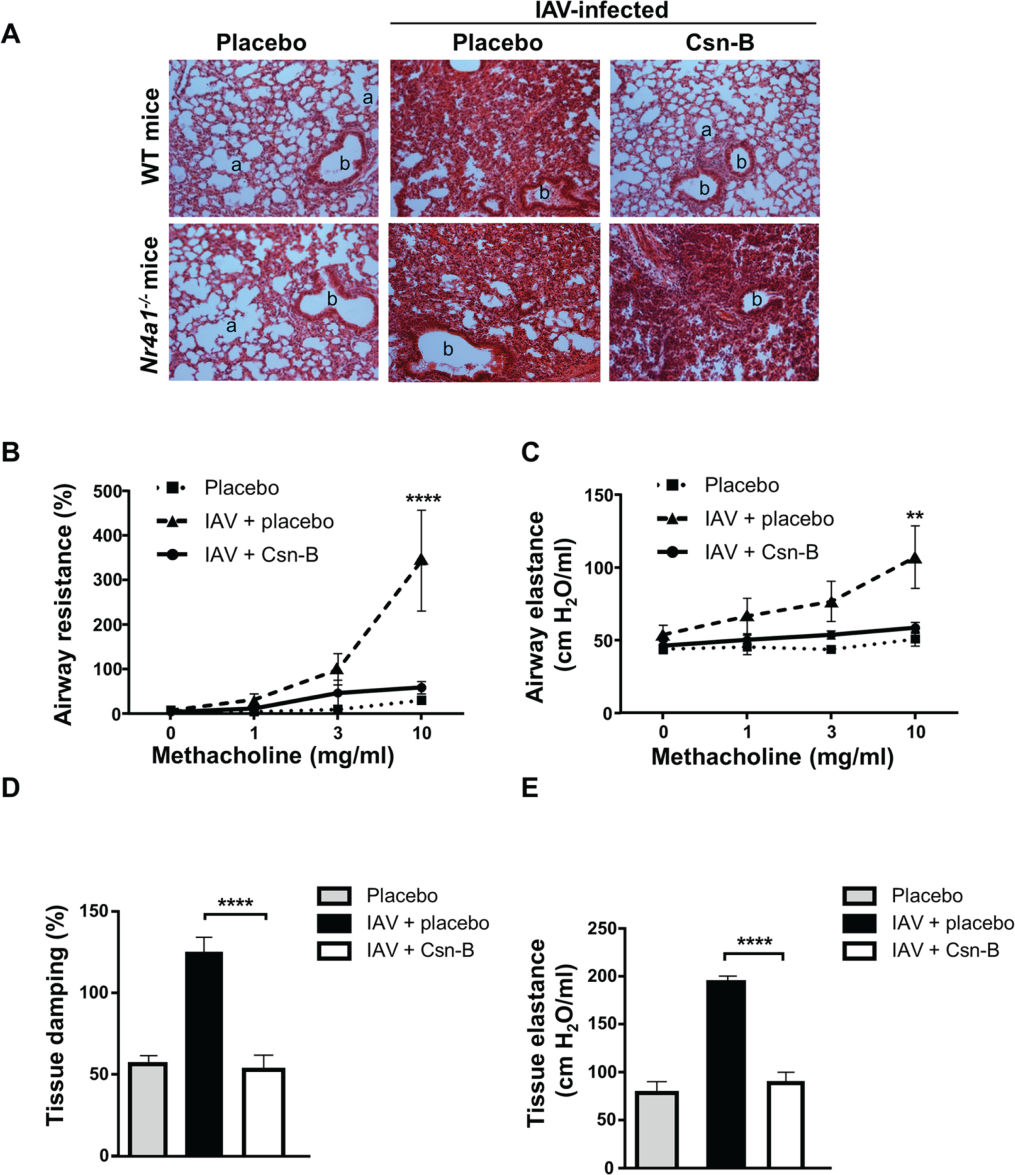
Treatment with cytosporone B improves lung structure and function in mice infected with Influenza A virus. **(A)** Haematoxylin and eosin stained of lung sections from not infected or IAV-infected (50 PFU) WT and *Nr4a1*^*-/-*^ mice, treated daily with placebo or Csn-B (5 mg/kg). Lungs were harvested at day 5 post-infection. Images are representative of two independent experiments (n = 3 mice/group). a: alveolar and b: bronchiolar structure (original magnification 100X). At day 5 pi., **(B)** airway resistance, **(C)** airway elastance, **(D)** tissue damping and **(E)** tissue elastance were measured (n = 6 mice/groups) upon methacholine challenge. Whilst baseline elastance was measured prior to methacholine administration using the FlexiVent apparatus. (**B, C**) Results are presented as mean ± SEM. **p ≤ 0.01 and ****p ≤ 0.0001 as compared to IAV + Csn-B. Differences in groups were determined using Two-Way ANOVA followed by Dunnett post-hoc test. (**D, E**) Results are presented as mean ± SEM. ****p ≤ 0.0001 as compared to IAV + Csn-B. Differences in groups were determined using One-Way ANOVA followed by Dunnett post-hoc test.

It is well known that IAV infection can affect structures and functions of the lungs [28, 29]. This prompted us to assess whether treatment of IAV-infected mice with Csn-B can reduce lung inflammation and consequently, improve pulmonary function. Histological examination revealed a marked decrease in leukocyte infiltration and a reduced bronchiolar wall thickening and alveolar obstruction in the lungs of Csn-B-treated IAV-infected WT mice as compared to placebo groups (Fig 2A). As expected, the effects of Csn-B were strongly abrogated in *Nr4a1*^-/-^mice infected with IAV. To examine the ability of the treatment of Csn-B to restore lung functions during IAV infection, we have measured lung parameters including airflow resistance as well as elastance. To do so, baseline lung functions and responsiveness to a methacholine (MCh) challenge were monitored at day 5 pi., in tracheotomized and ventilated animals using the FlexiVent apparatus. Increasing doses of MCh were nebulized to induce airway responsiveness and changes in the whole respiratory system resistance, tissue damping and tissue elastance were compared in IAV infected mice treated with Csn-B or with a placebo.

Results obtained indicate that Csn-B treatment improve these different parameters compared to untreated IAV-infected mice (Fig 2B, 2C, 2D and 2E). To provoke a contraction of the airways and test the reactivity of the airway system, increasing doses of methacholine were administered. Airways functions were significantly improved as we can see a reduction of airway resistance and elastance in mice treated with Csn-B compared to untreated IAV-infected animals (Fig 2B and 2C). In addition, tissue damping and tissue elastance were tested prior methacholine administration. In pulmonary alveoli, we saw a reduction of tissue damping and tissue elastance in mice treated with Csn-B compared to untreated IAV-infected animals. Moreover, airway functions of mice treated with Csn-B were similar to control groups, suggesting that treatment fully protects against virus-induced airway and tissue hyperresponsiveness. These results indicated that administration of Csn-B exerts beneficial effects on lung functions of IAV infected mice.

### Treatment with cytosporone B increases production of type I interferon in lungs of IAV-infected mice

**Fig 3:**
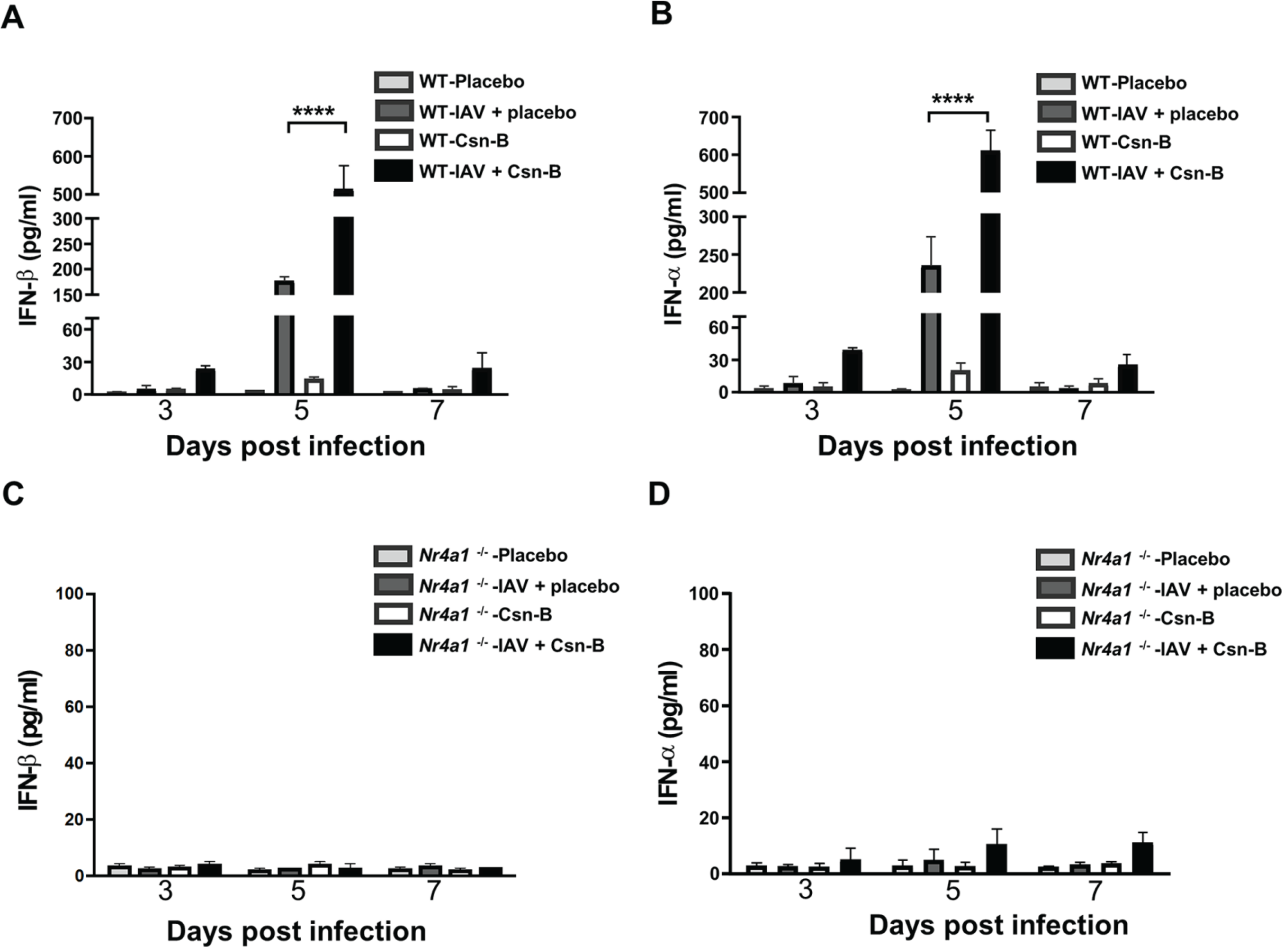
Treatment with cytosporone B increases production of type I interferons in bronchoalveolar fluids of IAV-infected mice. Levels of IFN-β and IFN-α production in bronchoalveolar fluids (BALs) from IAV-infected (**A-B)** WT and **(C-D)** *Nr4a1*^***-/-***^ mice (n = 4 mice/group), daily treated with placebo or Csn-B (5 mg/kg). BALs were collected at day 3, 5 and 7 post-infection. Results are presented as mean ± SEM of two independent experiments. Differences were determined using Two-Way ANOVA followed by Tukey post-hoc test. ****p ≤ 0.0001 as compared to indicated groups.

To further evaluate whether the effects of Csn-B treatment of lung viral loads correlate with the production of type I interferons (IFNs), we have measured synthesis of IFNβ/α in BALs of IAV-infected WT and *Nr4a1*^*-/-*^ mice treated with Csn-B. At day 5pi., we observed that Csn-B treatment significantly increased both IFN-β and IFN-α production in BALs of IAV-infected WT mice as compared to placebo groups (Fig 3A and 3B). Treatment of naive mice with Csn-B induces very low levels of IFNs. As expected, effects of Csn-B treatment on the synthesis of IFNs in BALs of IAV-infected mice were strongly abolished in mice deficient for NR4A1 (Fig 3C and 3D). Indeed, secretion of both IFN-β and -α was reduced by more than 96% in infected mice deficient for NR4A1, supporting that Csn-B and consequently NR4A1 contribute to the IFN response to IAV infection. Similar profiles of type 1 IFN synthesis were measured in sera of IAV-infected WT mice treated with Csn-B (data not shown). Together, these results indicate that Csn-B treatment potentiates IFN secretion induced by IAV infection.

### Selective depletion of AMs abrogates effects of Csn-B on IAV infection

**Fig 4:**
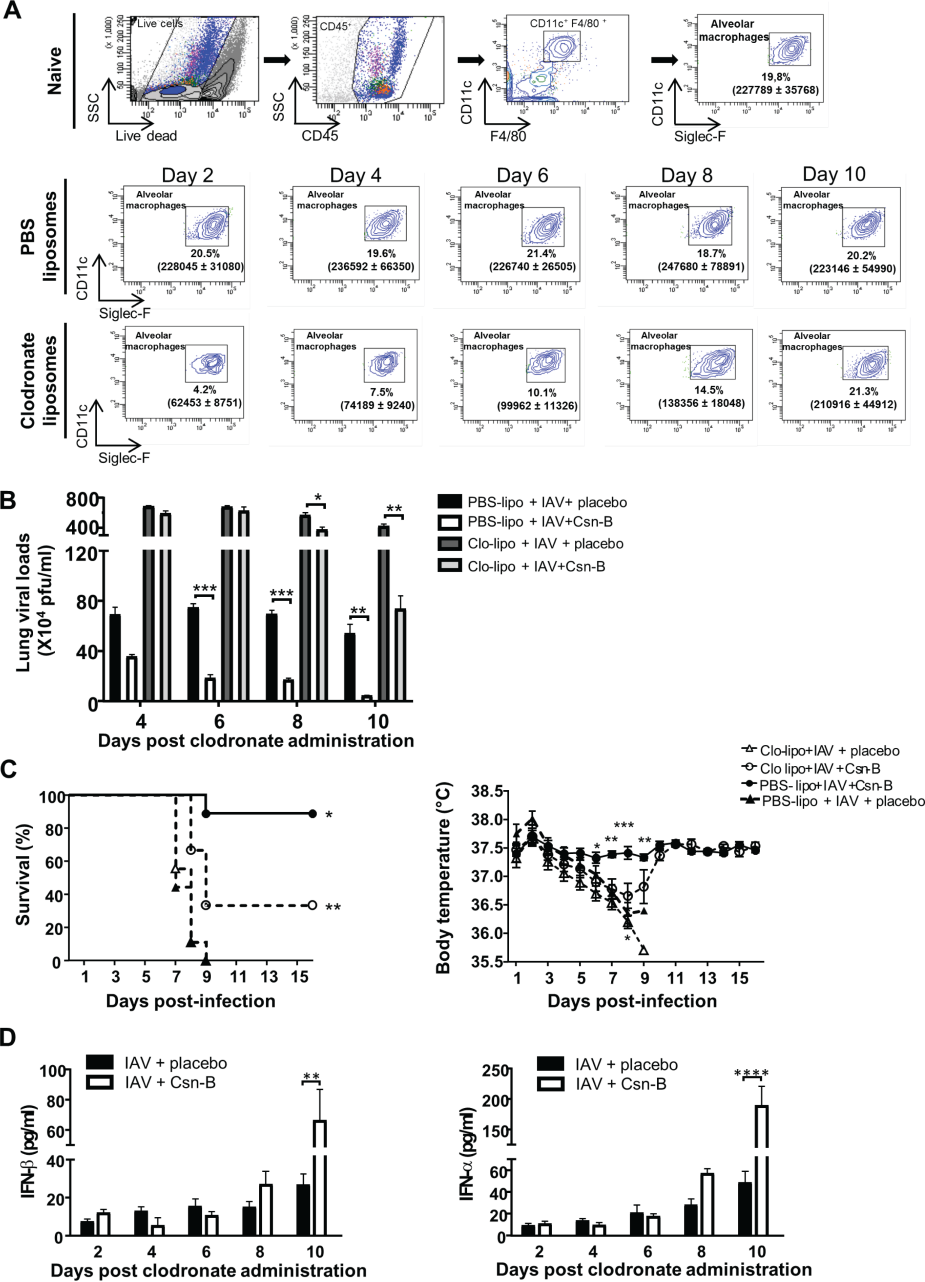
Selective depletion of alveolar macrophages reduces effects of Cytosporone B on IAV infection. **(A)** Representative gating strategies showing alveolar macrophages (AMs) in naive mice after PBS or clodronate liposome administration. AMs were gated as CD45^+^, CD11b^-^, CD11c^high^, F4/80^+^, Siglec-F^high^ cells in lungs of naive and PBS or clodronate liposomes treated WT mice. Data are presented as the frequencies (%) of AMs on CD45^+^ cells and true count values of AMs at indicated time following PBS or clodronate liposome administration. **(B)** Lung viral loads were assessed in IAV-infected WT mice administered with PBS-lipo (control) or Clo-lipo and daily treated with placebo or Csn-B (5 mg/kg ip.). Lungs were harvested at day 4, 6, 8 and 10 post-clodronate administration. Results are presented as mean ± SEM of two independent experiments (n = 4 mice/group). Differences were determined using Two-Way ANOVA followed by Dunnett post-hoc test. *p ≤ 0.05, **p ≤ 0.01 and ***p ≤ 0.001 as compared to indicated groups. **(C)** WT mice injected with PBS-lipo or Clo-lipo (n = 9/group) were infected with IAV (lethal dose of 3000 PFU in.) and daily treated for 16 days with either placebo or Csn-B (5 mg/kg). Survival and body temperature were monitored daily. For survival data, differences were analysed using a log rank test. *p ≤ 0.05 as compared to Clo-lipo + IAV + Csn-B. ** *p* ≤0.01 as compared to Clo-lipo + IAV + placebo. For body temperature data, differences were analysed using Two-Way ANOVA followed by Dunnett post-hoc test. *p ≤ 0.05 and **p ≤ 0.01 and ***p ≤ 0.001 as compared to Clo-lipo + IAV + Csn-B. **(D)** Levels of IFN-β and IFN-α were assessed in BALs of IAV infected mice (50 PFU) treated with placebo or Csn-B at indicated time following Clo-lipo administration. Results are presented as mean ± SEM of two independent experiments (n = 4 mice/groups). Differences were determined using Two-Way ANOVA followed by Dunnett post-hoc test. **p ≤ 0.01 and ****p ≤ 0.0001 as compared to indicated groups.

AMs have been shown to actively contribute to viral clearance and to represent an important producer of type 1 IFN upon IAV infection [18, 30, 31]. To evaluate whether AMs may respond to Csn-B treatment, we have first compared expression of NR4A1 mRNA in AMs of mice treated with Csn-B or a placebo. Our results indicate that Csn-B treatment markedly increases NR4A1 expression in AMs (S1B Fig). Next, to further evaluate the contribution of AMs in Csn-B action, we selectively depleted AMs using clodronate liposomes as detailed [18]. Dendritic cells remained unaffected in clodronate-treated mice ([18] and S2 Fig). Treatment of naive mice with clodronate-liposomes strongly reduced levels of AMs by day 2 which started to re-emerge by day 6 post-clodronate administration (Fig 4A). Deletion of AMs resulted in increased lung viral loads and mortality as well as an increased morbidity and disease severity as reflected by a reduced body temperature compared to the WT controls (Fig 4B and 4C). In addition, infected mice treated with clodronate-liposomes showed a strong decrease of type 1 IFN which began to be detectable at day 8 when restoration of lung AMs was close to normal levels (Fig 4D). Similarly, a decrease in leukocyte infiltration and alveolar obstruction and a reduced production of inflammatory cytokines TNF-α and IL-6 (S3 Fig) were found to coincide with the reappearance of AMs. These results support the conclusion that AMs are an important cell population activated in the lungs following treatment with Csn-B to control IAV infection.

### IRF3 and IRF7 contribute to Csn-B-induced IFN secretion in response to IAV infection

**Fig 5:**
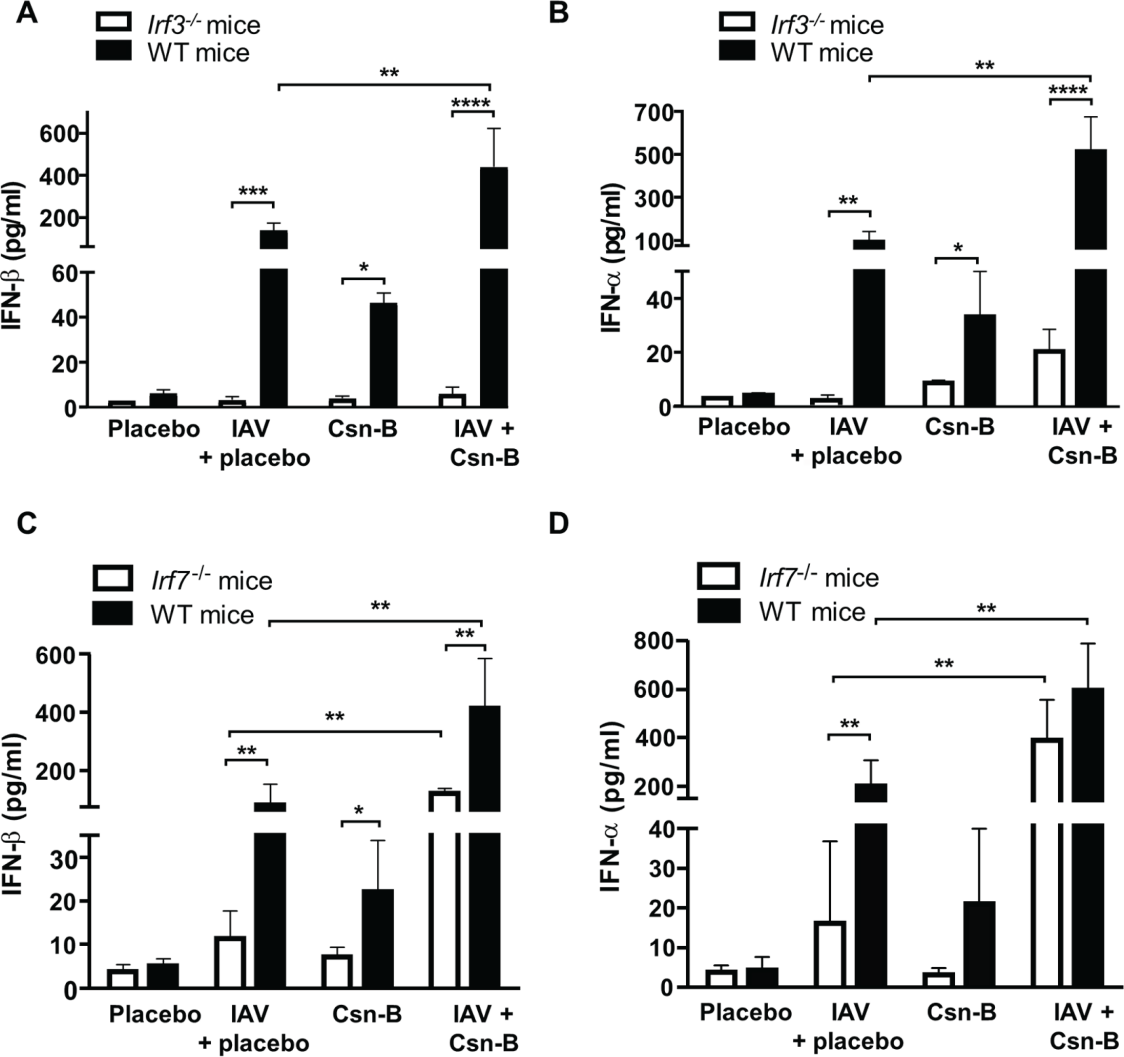
IRF3 and IRF7 contribute to Cytosporone B-induced IFN secretion in response to IAV infection. WT, *Irf3*^*-/-*^ *and Irf7*^*-/-*^ mice were infected with IAV (50 PFU) and daily treated with placebo or Csn-B (5 mg/kg). Levels of **(A-C)** IFN-β and **(B-D)** IFN-α production in BALs from IAV-infected WT, *Irf3*^***-/-***^ and *Irf7*^***-/-***^ mice, daily treated with placebo or Csn-B (5 mg/kg). BALs were collected at day 5 post-infection. Levels of IFN-β and IFN-α production in BALs of *Irf3*^***-/-***^ and *Irf7*^***-/-***^ mice were negligible at day 3 and 7 post-infection (data not shown). Results are presented as mean ± SEM of two independent experiments (n = 4 mice/groups). Differences were determined using Two-Way ANOVA followed by Tukey post-hoc test. *p ≤ 0.05 **p ≤ 0.01 ***p ≤ 0.001 and ****p ≤ 0.0001 as compared to indicated groups.

Interferon regulatory factors (IRF) 3 and 7 are recognized as key regulators of type 1 IFN in response to viral infection [32, 33]. To evaluate whether deficiency of IRF3 and IRF7 may affect action of Csn-B, *Irf3*^*-/-*^ and *Irf7*^*-/-*^ mice were infected with IAV and daily treated with Csn-B. At day 5 post-infection, production of IFNβ/α was measured in BAL fluids of WT and deficient mice. Our results show that secretion of IFN-β and -α induced following IAV infection was markedly reduced in BALs of *Irf3*^*-/-*^ infected mice compared to the WT controls, suggesting that IRF3 is required for Csn-B to potentiate production of IFNs (Fig 5A and 5B). On the other hand, when using *Irf7*^*-/-*^mice, effects of Csn-B treatment on IFN synthesis were slightly affected compared to the IAV-infected controls (Fig 5C and 5D). Together, these data suggest that IRF3 is essential to the action of Csn-B, but IRF7 plays rather a modest role. As expected, a combination of IRF3 and IRF7 contributes to activate the production of type 1 IFN in response to IAV infection [34–36].

**Fig 6:**
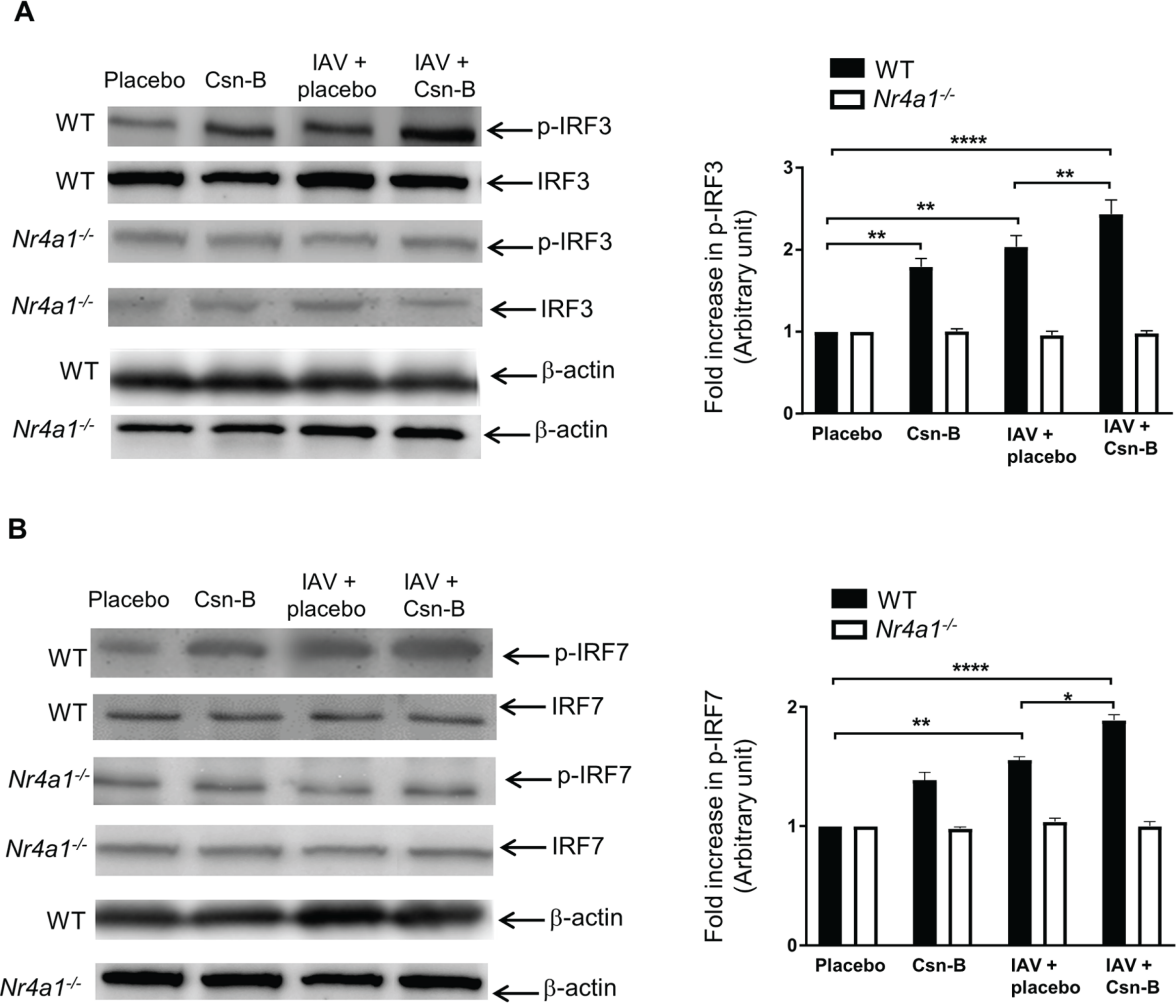
Phosphorylation of IRF3 and IRF7 are increased following Cytosporone B treatment of IAV-infected mice. WT and *Nr4a1*^*-/-*^ mice were infected with IAV (50 PFU) and daily treated with placebo or Csn-B (5 mg/kg). Lungs were harvested at day 5 post-infection. **(A)** Immunoblots of phosphorylated-IRF-3 on serine 396 (p-IRF3), IRF3 and **(B)** phosphorylated IRF7 on serine 471/472 (p-IRF7) and IRF7 were performed on protein extracted from lung homogenates of IAV-infected mice treated with placebo or Csn-B. Data are representative of two independent experiments (n = 3 mice/groups). ß-actin was used as loading control. Fold increase in protein expression is expressed relative to the placebo group. Data are representative of two independent experiments. Differences were determined using Two-Way ANOVA followed by Tukey post-hoc test. *p ≤ 0.05 **p ≤ 0.01 and ****p ≤ 0.0001 as compared to indicated groups.

To further determine if activation of IFN gene by Csn-B is dependent on IRF3 and IRF7, we performed western blot analysis on lung homogenates of IAV-infected mice treated or not with Csn-B. As expected, IAV infection leads to an increase of the phosphorylation levels of IRF3 (Fig 6A) [36]. Administration of Csn-B to naive mice clearly enhances the phosphorylation of IRF3 and potentiates phosphorylation levels induced by IAV infection, supporting a plausible link between NR4A1 and IRF3 in the activation of IFN genes. This correlates with the reduced levels of phosphorylation measured in lungs of mice lacking functional NR4A1 (Fig 6A). We have repeated the same approach to determine whether IRF7 is activated upon treatment with Csn-B. In contrast to IRF3, Csn-B treatment has a modest effect on IRF7 phosphorylation (Fig 6B) but increases its phosphorylation levels in the presence of IAV, suggesting that Csn-B may amplify the production of IFN synthesis through a mechanism mainly requiring the involvement of IRF3 instead of IRF7.

## Discussion

While the transcription factor NR4A1 is now recognized to be involved in the regulation of inflammatory processes and immune response, its contribution in host defense against pathogens is largely unknown [1, 2]. We have evaluated whether cytosporone B (Csn-B), a specific agonist of NR4A1 [9], can counter IAV infection in mice. AMs are well known to actively participate in viral clearance. Indeed, AMs are potent producers of type 1 IFN in response to IAV and their depletion was found to result in increased lung viral loads and to a decreased production of IFNs [31]. Our results demonstrated that AMs are sensitive to the action of Csn-B as reflected by the increase levels of NR4A1 mRNA in these cells. In addition, protective effects of Csn-B on morbidity and mortality were markedly reduced by the selective depletion of AMs. Similarly, the production of IFNs was also markedly affected by the depletion of AMs which significantly increases when a fraction of AMs began to be restored. However, we must also consider that other resident cells in the lungs like macrophages and dendritic cells expressing NR4A1 may secrete type 1 IFN following the stimulation with Csn-B. Nevertheless, AMs remain potent producers of IFNs [30, 37] and our data indicate that this cell population plays a significant role in the antiviral capacities of Csn-B to control IAV infection.

Our results show that the secretion of type 1 IFN in response to IAV infection is a mechanism that likely contributes to the antiviral activity of Csn-B. In addition, such potentiation of IFN synthesis by Csn-B was found to mainly require the phosphorylation of IRF3 and to a lesser extent, the activation of IRF7. Unlike IRF3 which is constitutively expressed in resting cells, IRF7 is activated upon stimulation of cytosolic innate receptors to potentiate type 1 IFN gene induction. Thus, IRF3 is essential for the induction of IFN-β as an early response to virus infection and IRF7 leads to the amplification signals of IFN-α genes. We believe that although Csn-B may activate both IRF3 and IRF7, it might activate signaling events that lead to the phosphorylation of IRF3 more efficiently than IRF7, a process that may contribute to counter IAV in the early stages of infection. This hypothesis could explain our data showing that the production of IFNs in IAV-infected mice deficient for IRF7 was much less affected than in *Irf3*^*-/-*^ mice. Furthermore, Csn-B being a specific agonist of NR4A1, this transcription factor might have an impact on IRF3 and/or IRF7 phosphorylation. This is also plausible since previous studies have demonstrated the regulatory role of NR4A1 in controlling NF-κB activity and consequently expression of pro-inflammatory target genes [38]. A molecular mechanism activated by NR4A1 is the inhibition of NF-κB/p65 binding to κB binding sites on gene promoters [39]. Based on these interactions between NR4A1 and NF-κB, it is reasonable to believe that once activated by Csn-B, NR4A1 might increase phosphorylation of IRF3 and consequently activate the synthesis of type 1 IFN. However, whether NR4A1 can directly induce the phosphorylation of IRF3 or whether “a bridge protein” is required to phosphorylate IRF3 is not yet elucidated and additional experiments are required to address this question.

Our study also showed that there is a significant improvement of lung integrity attributable to a reduction of leukocyte infiltration in Csn-B-treated IAV-infected WT mice as compared to the placebo groups. This effect is dependent on NR4A1, as improvement of lung integrity was abrogated in *Nr4a1*^*-/-*^ mice. This correlates with a gain of lung function parameters such as resistance and elastance. The beneficial effects of Csn-B treatment on pulmonary function during IAV infection could however, involve different mechanisms that can contribute to reduce excessive inflammation and to restore lung homeostasis. First, following its activation by Csn-B, NR4A1 can prevent translocation of NF-κB through the activation of IκB, thereby reducing the secretion of inflammatory response [3, 38, 40]. This hypothesis is in line with our study showing that treatment of IAV-infected mice with Csn-B significantly reduces the concentration of inflammatory cytokines like TNF-α and IL-6 in lungs of infected mice. Secondly, stimulation with Csn-B can increase expression of NR4A1 in several cell types including patrolling Ly6C^low^ monocytes that are known to have the capacity to polarize into anti-inflammatory M2-type macrophages within the infected tissues [6, 9, 41, 42]. Thirdly, we believe that Csn-B treatment, by increasing expression of NR4A1 in immune cells contribute to control excessive lung inflammation in infected mice by reducing production of inflammatory cytokines and by preventing the prolonged neutrophil influx in the late stage of infection [43]. Another biological function associated with NR4A1 to control inflammation resides in its ability to regulate T lymphocyte activation [44] which expresses NR4A1. In fact, using a murine model of arthritis, we have demonstrated that treatment of arthritic mice with Csn-B results in an increase in circulating Treg cells which contributes to reduce joint inflammation [45]. It is plausible that similar effects can occur in lungs of infected mice treated with Csn-B. Therefore, in light of the results obtained, we can postulate that treatment with Csn-B (and consequently activation of NR4A1) controls the spread of viral infection and also protects from morbidity and from excessive lung inflammation induced by IAV infection.

**Fig 7:**
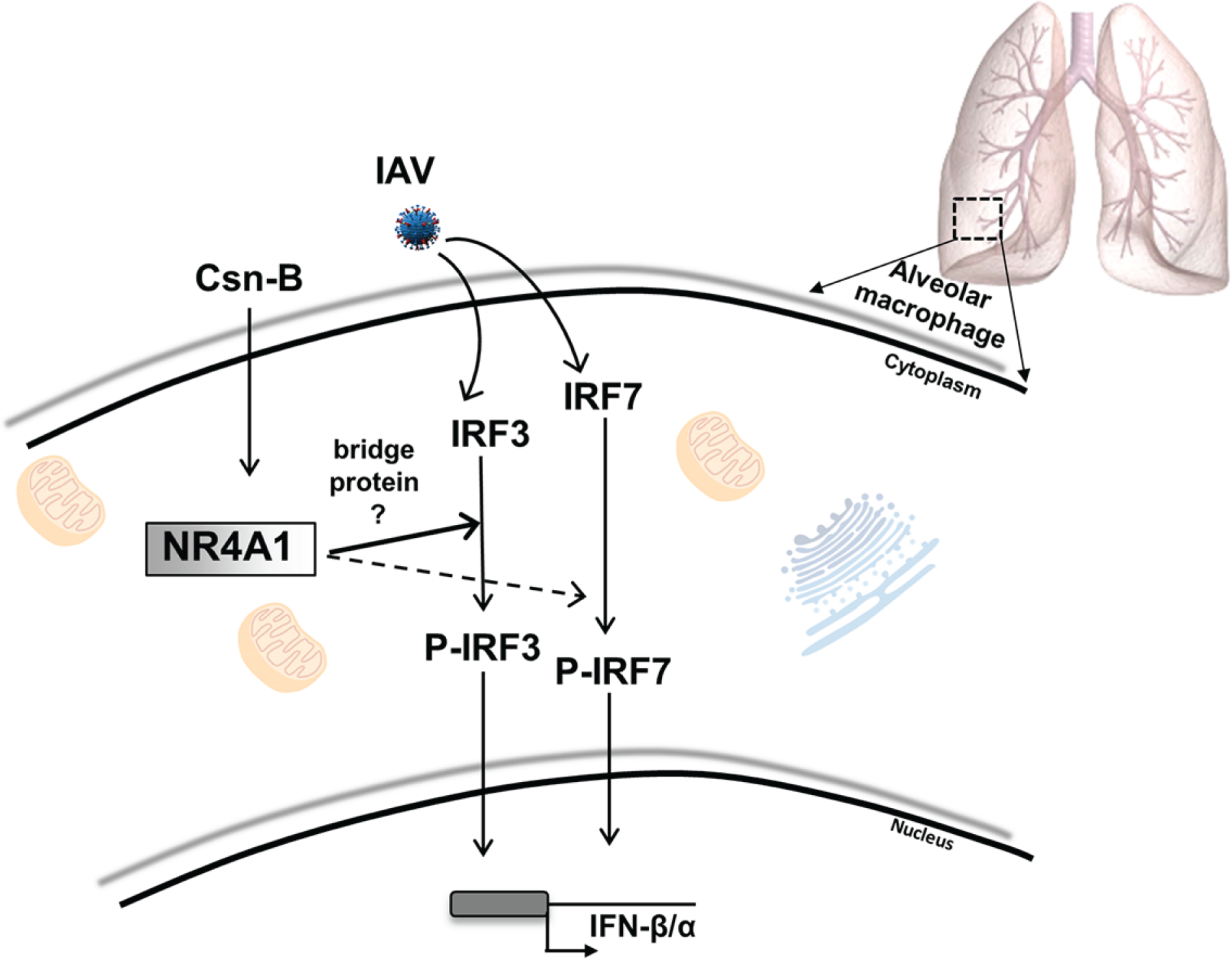
Hypothetical scenario of the effects of Csn-B on the production of type 1 IFNs in bronchoalveolar lavages of mice infected with IAV. Influenza A virus infection of WT mice induces phosphorylation of IRF3 and IRF7 transcription factors. As primary mechanism, treatment with Csn-B, an agonist of NR4A1, potentiates phosphorylation of IRF3 (solid line) and then, to a lesser extent, phosphorylates IRF7 (dashed line). These effects consequently increase production of type 1 IFNs in alveolar macrophages.

In light with our findings, we propose a mechanism through which after Csn-B treatment, cytoplasmic NR4A1 potentiates innate immune response against IAV infection (Fig 7). Csn-B, a NR4A1 agonist, enhances the IAV-mediated phosphorylation of IRF3 and IRF7 which, in turn, potentiate the production of type 1 IFN in alveolar macrophages. However, whether NR4A1 can directly induce phosphorylation of IRF3 and IRF7 or require “a bridge protein” is not yet elucidated.

In conclusion, we have demonstrated that activation of the transcription factor NR4A1 by the agonist Csn-B markedly stimulates clearance of IAV in lungs of infected mice and restores respiratory functions. The stimulation of type 1 IFN synthesis by AMs appears to be a key mechanism associated to Csn-B to control IAV infection and to protect from excessive inflammatory lung injuries following infection. Through its actions on IRF3, our results argue that Csn-B can activate host defense against respiratory pathogens and maintain lung homeostasis. Hence, a better knowledge of the interactions of NR4A1 with the immune response should provide insights for the elaboration of new therapeutic strategies to control respiratory infections.

## Supporting information

S1 Fig Cytosporone B specifically increases NR4A1 mRNA expression in lungs of WT mice. (**A**) NR4A1, NR4A2 and NR4A3 mRNA levels were assessed by RT-PCR in lungs of WT mice daily treated with placebo or Csn-B (5 mg/kg) for 5 days. GAPDH was used as internal control. Data are representative of two independent experiments (n = 3 mice/group). (**B**) NR4A1 mRNA levels were assessed by qRT-PCR in BALs of WT mice treated with placebo or Csn-B (5 mg/kg) for 45 minutes. GAPDH was used as internal control. Data are representative of two independent experiments (n = 3 mice/group). Differences were determined using a Mann-Whitney test. * *p* ≤ 0.05.
(EPS)

S2 Fig Clodronate liposome administration has no effect on lungs dendritic cells. Representative gating strategies of lung dendritic cells following PBS or clodronate liposome administration. Autofluorescent cells were excluded by gating on CD45^+^and FITC^-^. Thereafter, dendritic cells were identified as CD11c^high^ and MHCII^+^ cells in lungs of WT mice at day 2 following PSB or clodronate liposome administration. Data are presented as absolute numbers of dendritic cells and are representative of two independent experiments (n = 3 mice/group).
(EPS)

S3 Fig Alveolar macrophages are involved in the cytosporone B-induced improvement of lung structure and integrity in mice infected with IAV. (**A**) Haematoxylin and eosin stained lung sections from not infected or IAV-infected (sublethal dose of 50 PFU in.) WT mice daily treated with placebo or Csn-B (5 mg/kg). One day prior to IAV infection clodronate-liposome was administered (in.) in infected mice in order to deplete alveolar macrophages. Lungs were harvested at day 2 (depleted AMs) and 10 (restoration of AMs) post clodronate administration. Images are representative of two independent experiments (n = 3 mice/groups). a: alveolar and b: bronchiolar structure. (**B**) Levels of TNF-α and IL-6 were assessed in lung homogenates of IAV infected mice daily treated with placebo or Csn-B. Lungs were harvested at day 10 post-clodronate administration. Results are presented as mean ± SEM of two independent experiments (n = 4 mice/groups). Differences were determined with a One-way ANOVA followed by a Tukey post-hoc test. *p ≤ 0.05 as compared to indicated groups.
(EPS)
